# Mathematical Modeling Reveals Kinetics of Lymphocyte Recirculation in the Whole Organism

**DOI:** 10.1371/journal.pcbi.1003586

**Published:** 2014-05-15

**Authors:** Vitaly V. Ganusov, Jeremy Auerbach

**Affiliations:** 1 Department of Microbiology, University of Tennessee, Knoxville, Tennessee, United States of America; 2 Department of Mathematics, University of Tennessee, Knoxville, Tennessee, United States of America; Emory University, United States of America

## Abstract

The kinetics of recirculation of naive lymphocytes in the body has important implications for the speed at which local infections are detected and controlled by immune responses. With a help of a novel mathematical model, we analyze experimental data on migration of ^51^Cr-labeled thoracic duct lymphocytes (TDLs) via major lymphoid and nonlymphoid tissues of rats in the absence of systemic antigenic stimulation. We show that at any point of time, 95% of lymphocytes in the blood travel via capillaries in the lung or sinusoids of the liver and only 5% migrate to secondary lymphoid tissues such as lymph nodes, Peyer's patches, or the spleen. Interestingly, our analysis suggests that lymphocytes travel via lung capillaries and liver sinusoids at an extremely rapid rate with the average residence time in these tissues being less than 1 minute. The model also predicts a relatively short average residence time of TDLs in the spleen (2.5 hours) and a longer average residence time of TDLs in major lymph nodes and Peyer's patches (10 hours). Surprisingly, we find that the average residence time of lymphocytes is similar in lymph nodes draining the skin (subcutaneous LNs) or the gut (mesenteric LNs) or in Peyer's patches. Applying our model to an additional dataset on lymphocyte migration via resting and antigen-stimulated lymph nodes we find that enlargement of antigen-stimulated lymph nodes occurs mainly due to increased entrance rate of TDLs into the nodes and not due to decreased exit rate as has been suggested in some studies. Taken together, our analysis for the first time provides a comprehensive, systems view of recirculation kinetics of thoracic duct lymphocytes in the whole organism.

## Author Summary

It is has been well established that lymphocytes, major cells of the adaptive immune system, continuously recirculate between secondary lymphoid tissues in the body such as lymph nodes, spleen, and Peyer's patches. Yet, the kinetics of lymphocyte entrance into and more importantly, residence times in these tissues still remain incompletely determined. Using previously published experimental data and a novel mathematical model we address this problem and estimate the rates of lymphocyte entrance into and exit from major secondary lymphoid tissues. We find that lymphocytes on average must pass via vasculature of the lung or liver 20 times before they migrate to one of the secondary lymphoid tissues. Despite small size of capillaries in the lung and liver, we predict that on average lymphocytes spend less than 1 minute in the vasculature of these organs. Furthermore, we estimate that lymphocytes spend on average 2.5 hours in the spleen and 10 hours in the lymph nodes or Peyer's patches. Our mathematical modeling for the first time provides a quantitative, systems view of lymphocyte recirculation in the whole organism.

## Introduction

Lymphocytes, cells of the adaptive immune system, continuously recirculate between multiple tissues in the body [1]–[3]. Naive, antigen-unexperienced lymphocytes are traditionally thought to recirculate mainly between blood, lymph, and secondary lymphoid organs such as spleen, lymph nodes, and Peyer's patches [2], [4], [5]. Effector and memory lymphocytes can access nonlymphoid tissues such as the brain, skin, lung, vaginal tract, salivary gland, and gut epithelium [6]–[15]. Although receptors and their ligands regulating entrance of lymphocytes into various lymphoid or nonlymphoid tissues have been thoroughly studied [16]–[20], how quickly lymphocytes can enter a particular tissue and how long they will stay in that tissue remains incompletely understood. Furthermore, how lymphocyte recirculation kinetics depends on the cell type (naive, memory, or effector T or B lymphocyte), the tissue (e.g., lymphoid or nonlymphoid), localization in the tissue (e.g., vasculature or parenchyma), and status of the host (e.g., uninfected or infected) remains understudied.

The number of lymphocytes found in a particular tissue results from several different processes: entrance of lymphocytes in the tissue, lymphocyte proliferation in the tissue, lymphocyte death in the tissue, and lymphocyte exit from the tissue [6]. Change in either of these processes, for example, during an infection, will change the number of lymphocytes residing in the tissue. Yet, which of these four processes actually regulate cell abundances in various tissues under different conditions is incompletely understood [6], [21], [22]. For example, it has been suggested that naive T cells have the ability to enter peripheral tissues such as gut lamina propria or the brain [6], [23], [24] but what regulates low recovered numbers of naive T cells in these tissues (e.g., a low entrance rate into the tissue or a high death rate in the tissue) remains unclear.

Patterns and kinetics of lymphocyte migration via secondary lymphoid tissues have been studied extensively several decades ago using various *ex vivo* and *in vivo* experimental techniques including lymph node or thoracic duct cannulation [6], [25]–[27]. Yet, interpretation of the experimental data obtained in such kinetic studies remained semi-quantitative. Transit times via a particular lymph node (e.g., popliteal or inguinal) have been estimated in large animals such as sheep or pigs by transferring labeled lymphocytes intravenously into the animal and measuring the rate of exit of labeled lymphocytes into efferent lymph of the cannulated node [1], [4], [28]–[33]. These experiments showed that the peak of labeled cell exit during cannulation occurs 20–30 hours post lymphocyte transfer with many lymphocytes exiting the lymph node for several days after the transfer. It has been implicitly assumed that the mode (or the average) of this distribution represents the average residence time of lymphocytes in lymph nodes. Very recently, a novel mathematical model has been developed to estimate the average transit time of labeled lymphocytes via individual lymph nodes from such cannulation experiments suggesting that blood-derived lymphocytes spend on average 30 hours in sheep lymph nodes [33].

Thoracic duct cannulation has been used in mice and rats to study kinetics of lymphocyte migration from the blood to the lymphatic duct lymph [34]–[40]. Such experiments reported the change in the rate of exit of adoptively transferred labeled lymphocytes via the thoracic duct over time. Depending on the study, the animal species, and the type of lymphocytes used for transfer, the peak of lymphocyte exit from the thoracic duct ranged from 15 to over 24 hours [25], [35]. Similarly to the data on cannulation of individual lymph nodes, the mode or the mean of the distribution of exit rates of labeled lymphocytes during thoracic duct cannulation has been interpreted to represent the average residence time of lymphocytes in lymph nodes [18], [19], [30], [41]. Mathematical models that assumed that lymphocytes spend 6 hours in the spleen and 20 hours in lymph nodes of rats could explain kinetics of exit of adoptively transferred labeled lymphocyte during thoracic duct cannulation [42]–[44].

More recently, the average residence time of naive and memory T lymphocytes in major lymph nodes in mice has been estimated using mathematical models [45], [46]. In these studies naive or memory T lymphocytes were adoptively transferred into syngenic hosts and later, lymphocyte entry into lymph nodes was blocked using antibodies against specific receptors such as L-selectin or LFA-1 and 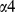. The rate of decline of the number of donor lymphocytes in various lymph nodes was used to calculate the average residence time of lymphocytes in the lymph nodes [45], [46]. In particular, Mandl *et al*. [46] estimated the average residence time of naive CD4 T cells in peripheral and mesenteric LNs to be 12.2 and 9.6 hours, respectively, and that of naive CD8 T cells to be 21.2 and 17.0 hours respectively. In comparison, Harp *et al*. [45] found that both naive and memory CD8 T cells specific to lymphocytic choriomeningitis virus (LCMV) spend on average 8.4 hours in lymph nodes. Another recent study using photoconvertable protein-expressing mice (KikGR) estimated the half-life time of naive B cells in Peyer's patches of mice to be 10 hours [47].

Only a few studies investigated lymphocyte migration via other secondary lymphoid tissues such as the spleen. One study used *ex vivo* isolated rat spleens perfused with labeled lymphocytes. Based on the kinetics of lymphocyte loss from and return in the artificial circulation the average residence time of lymphocytes in the spleen was proposed to be around 5–6 hours [48]–[51]. A mathematical modelling study provided estimates of lymphocyte migration via the red and white pulp but did not estimate the overall lymphocyte transit time via the spleen [52].

The kinetics of migration of lymphocytes via vasculature of nonlymphoid organs such as the lung and liver remain poorly understood. Several lines of indirect evidence suggests that lymphocytes may spend tens of minutes to hours in the lung vasculature. First, the diameter of the capillaries in the lung (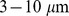 in humans) is similar to or smaller than the size of a lymphocyte (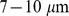 in humans), suggesting that many lymphocytes may be delayed while migrating via the lung vasculature [53]–[55]. Second, recent studies found that the majority of memory CD8 T lymphocytes isolated from a perfused murine lung following LCMV infection are in fact associated with lung capillary bed [56]. Third, transfer of *in vitro* activated lymphocytes to syngenic animals (mice and monkeys) leads to a long retention of transferred cells in the lung [57]–[59]. Finally, studies perfusing *ex vivo* isolated swine lungs demonstrated a relatively long average residence time of leukocytes in the lung [60]. Studies on migration of radioactively labeled neutrophils in several mammalian species suggest that the majority of neutrophils are retained during passage via the lung [61]–[63]. A recent study also suggested that naive CD4 T lymphocytes are retained in the lung for substantial periods of time [64]. Overall, previous studies indicate that under normal, uninflammatory conditions lymphocytes may spend substantial amount of time while traveling via the capillary bed of the lung. We have found no studies on the migration kinetics of lymphocytes via sinusoids of the liver.

Taken together, there is a wealth of experimental data on migration of lymphocytes via major secondary lymphoid and some nonlymphoid tissues. Unfortunately, the most often cited estimates of lymphocyte residence times in such tissues [18], [19], [30], [41] have been obtained by qualitative techniques without the use of mathematical modeling. Such implicit calculations make it difficult to judge which assumptions have been made during calculations and whether provided estimates should be trusted. There are more recent mathematical modeling-based estimates of the lymphocyte residence times in lymph nodes but it remains unknown if these estimates can be used to provide a comprehensive view of recirculation kinetics of lymphocytes via all the major organs in mammals. In addition, such estimates have been obtained using different experimental systems and are for different types of lymphocytes. In this paper we analyze data from experiments involving transfer of ^51^Cr-labeled thoracic duct lymphocytes and measurements of accumulation and loss of the labeled cells in most tissues of rats [65]. Using a novel mathematical model of lymphocyte recirculation in the whole organism we, for the first time, provide estimates of the migration kinetics of thoracic duct lymphocytes to all major murine organs/tissues and average residence times of lymphocytes in these tissues in one modeling study.

## Materials and Methods

### Experimental data

**Figure 1:**
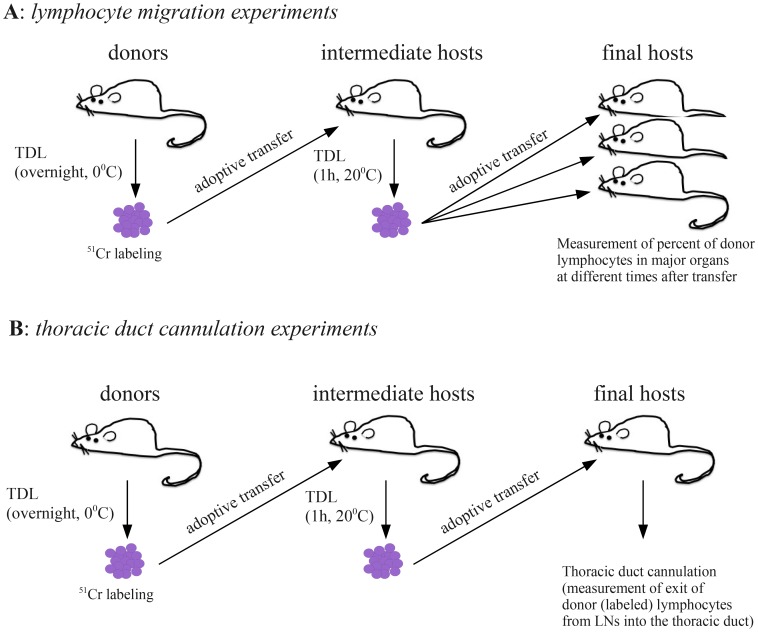
Two types of experiments performed in rats by Smith & Ford [65]. In the first set of experiments (migration data, panel A), thoracic duct lymphocytes (TDLs) were collected overnight from male donor rats by cannulation and labeled with sodium-[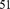
Cr] chromate. Labeled lymphocytes were passaged from blood to lymph *in vivo* by thoracic duct cannulation in an intermediate male. Collected lymphocytes were injected into female recipient rats and the percent of injected donor lymphocytes was measured in major lymphoid and nonlymphoid organs of the recipient rats. In the second set of experiments (cannulation data, panel B), TDLs were passaged via an intermediate host and then injected into the final recipients. Donor lymphocytes were counted during the thoracic duct cannulation of the recipient rats over the period of 45 hours.

Data on lymphocyte recirculation have been collected by Smith and Ford [65] in a series of experiments with AO rats (Figure 1). Lymphocytes were collected overnight from male donor rats by thoracic duct cannulation and labeled with sodium-[^51^Cr] chromate (16 hours, cells kept at 0°C). Prior work has demonstrated that long handling of thoracic duct lymphocytes (TDLs) at low temperatures influences ability of lymphocytes to migrate to lymphoid organs [65], [66]. Therefore, ^51^Cr-labeled lymphocytes were passaged from blood to lymph *in vivo* by thoracic duct cannulation in an intermediate male rat with minimal handling at room temperature for approximately 1 hour. Total radioactivity in collected lymphocytes was measured and then these lymphocytes were injected into female recipient rats. Thirteen organs of the recipients were removed at the following intervals after cell transfer: 1, 2, 5, 10 and 30 minutes, and 1, 2.5, 6, 9, 12, 15, 18, and 24 hours and the fraction of labeled cells recovered from a given organ was recorded (Figure 1A). The average percent of labeled TDLs was calculated from at least 5 rats [65].

The following organs were removed for counting of the ^51^Cr-labeled TDLs: six superficial cervical LNs, two to four deep cervical LNs, two to three coeliac LNs, the mesenteric LN chain excluding the ileocaecal LNs, the right popliteal LN (stimulated 3 days previously with 0.10 ml of a 10% suspension of sheep erythrocytes 3 days prior to cell transfer), the left popliteal LN (unstimulated), Peyer's patches, three samples of the small intestines with the Peyer's patches removed, the spleen, the left lung with large hilar vessels removed, samples of the liver, the right tibia and the mononuclear cells in the blood samples. Percent of labeled lymphocytes in all major lymph nodes except of mesenteric LNs were pooled together and denoted as subcutaneous LNs (SCLNs).

In a separate set of experiments the tempo of recirculation of TDLs from blood to the thoracic duct was evaluated (Figure 1B). ^51^Cr-labeled TDLs were were passaged through an intermediate host as described above and then injected into final recipients. Thoracic duct lymphocytes were collected from the recipient rats at 90 minute intervals over a 45 hour period and the percent of labeled TDLs exiting lymph nodes per hour via the thoracic duct was calculated. The data were exported from the original publication Smith & Ford [65] using GraphClick (www.arizona-software.ch/graphclick/) and Engauge Digitizer (digitizer.sourceforge.net/) software packages. Digitized data are available as supplemental material to this paper (Datasets S1, S2, S3) and at http://web.bio.utk.edu/Ganusov/mathematica/smith_i83-data.zip.

### Mathematical model

#### Basic assumptions

**Figure 2:**
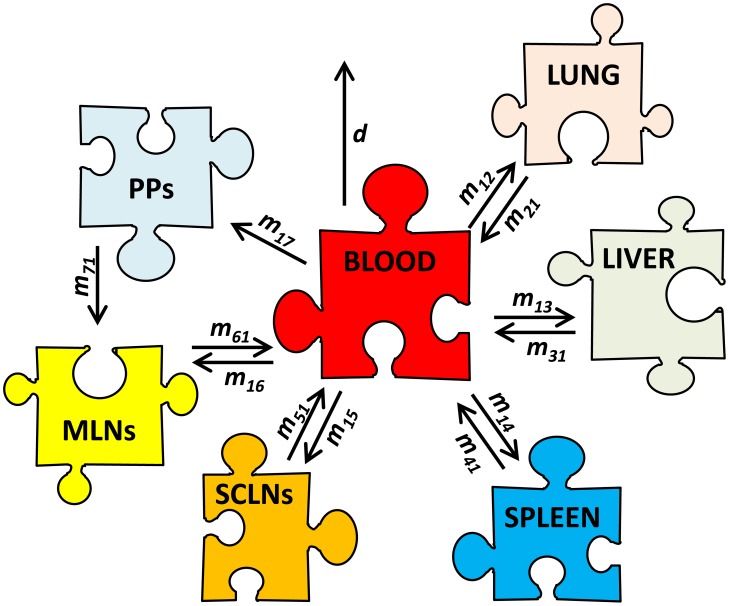
Cartoon representing migration of thoracic duct lymphocytes (TDLs) in rats. Blood is the main compartment that connects all tissues, and the rate of lymphocyte migration from the blood to other tissues is denoted as 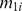 where 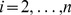 and 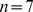. Cells leaving a particular organ return to the blood at a rate 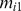 with the exception of lymphocytes in the Peyer's patches from which lymphocytes migrate to the mesenteric LNs at a rate 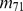 [67, p. 470]. In these experiments the total number of labeled cells declined over time (Figure 2 in Text S1), and therefore we allow for a constant removal rate of TDLs from the blood 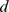 occurring due to death and/or migration of lymphocytes to other tissues that were not sampled. The percent of transferred lymphocytes was measured in the blood (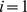), lung (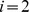), liver (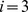), spleen (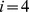), subcutaneous lymph nodes (SCLNs, 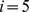), mesenteric lymph nodes (MLNs, 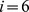), and Peyer's patches (PPs, 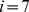). Lymphocytes exiting lymph nodes return to the blood via right lymphatic and left lymphactic (thoracic) ducts (see also Figure 1 in Text S1). The thoracic duct collects lymph from all mesenteric lymph nodes and from approximately half (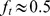) of subcutaneous lymph nodes [68]. Lymph from other subcutaneous lymph nodes (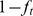) enters the blood via the right lymphatic duct [68], [69].

In the mathematical model we assume that the blood is the major compartment that delivers lymphocytes into other tissues and by exiting these tissues, lymphocytes return to the blood. The only exception to this rule is Peyer's patches as it is known that from Peyer's patches lymphocytes migrate into mesenteric lymph nodes ([67, p. 470]; Figure 2). It is important to mention that measurements of transferred cells in various organs included cells found in the vasculature (capillaries) and parenchyma (tissue). Therefore, our analysis delivers estimates of the average kinetics of TDLs via both vasculature and parenchyma. The model assumes that exit from nonlymphoid tissues and the spleen follows first order kinetics, and therefore, residence times in these tissues are exponentially distributed. Analysis of the data using models in which TDL residence times in these tissues are not exponentially distributed will be presented elsewhere.

#### Migration via lymph nodes

Exit lymphocytes from lymph nodes may not follow a simple exponential kinetics as there is an initial delay in the rate of recovery of labeled lymphocytes following thoracic duct cannulation [40], [65]. Therefore, to describe the dynamics of lymphocyte migration via lymph nodes and Peyer's patches we assume that these tissues consists of several (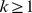) sub-compartments and in each sub-compartment the residence times are exponentially distributed at the same rate 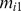. These different compartments 1) could be represented by different lymph nodes as it is known that lymph nodes often form chains and lymphocytes exiting from one lymph node enter another lymph node without returning to the blood [68]–[72]; or 2) different subcompartments within a given lymph node (e.g., cortex, paracortex, and medulla, [69]). We note that while there could be biological reasons for subcompartments, in our model these subcompartments should be rather considered as a mathematical method allowing for non-exponentially distributed residence times in the lymph nodes. In our case, these times are distributed in accord with gamma distribution. The average residence time of lymphocytes in the LNs or PPs is then given by 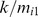. Lymphocytes exit LNs into the blood via two large lymphatic vessels, left and right lymphatic ducts [21], [68], [69], [73]. Thoracic (left lymphatic) duct collects lymphocytes exiting mesenteric lymph nodes and approximately half of lymphocytes exiting from subcutaneous lymph nodes into the blood [68], [73]. We let 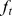 be the fraction of lymphocytes migrating to blood from the SCLNs via the thoracic duct. We expect that 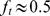 [68], [73].

#### Accumulation of dying cells in the liver

**Figure 3:**
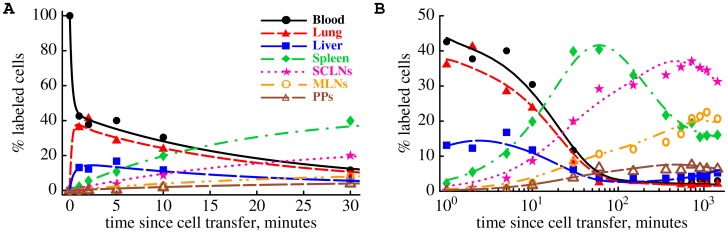
Mathematical model accurately predicts the hierarchy of recirculation of thoracic duct lymphocytes (TDLs) between major murine organs. 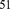
Cr-labeled TDLs were passaged via an intermediate host and then were transferred into syngenic rats (Figure 1A). The percent of transferred cells was measured at different times after cell transfer in major lymphoid and nonlymphoid murine organs and is shown by markers. We fit the mathematical model of lymphocyte recirculation (eqn. (1) – (6)) to these experimental data using nonlinear least squares; model fits are shown as lines. Plots are for the first 30 minutes of the experiment (A) or for the whole experiment (B, abscissa values are plotted on the log-scale). Parameter estimates of the model are given in Table 1. Different y-scales in panels A and B were used for clarity.

It has been established previously that ^51^Cr-labeled lymphocytes over time undergo apoptosis and dying cells tend to accumulate over time in the liver and/or kidneys [6]. To explain accumulation of labeled cells in the liver in our experimental data (Figure 3) we allow a fraction of labeled lymphocytes 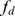 that are in the blood to migrate to the liver at a rate 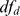 and remain in the liver as dying/dead cells. Here 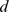 is the migration rate of lymphocytes from the blood to organs that were not sampled by Smith & Ford [65], and/or cell removal due to death (Figure 2).

With these assumptions the dynamics of thoracic duct lymphocytes in major murine organs is described by the following differential equations:
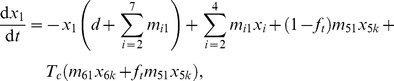
(1)




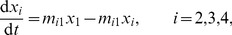
(2)




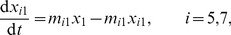
(3)




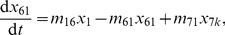
(4)




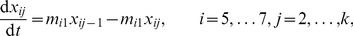
(5)




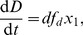
(6)where 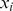 is the percent of labeled cells found in the blood (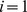), lung (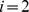), liver (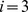), spleen (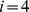), and 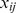 is the percent of labeled cells found in the 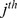 sub-compartment of the subcutaneous LNs (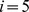), mesenteric LNs (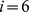), or Peyer's Patches (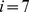) and 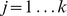. We denote 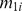 as the rate of lymphocyte migration from the blood to the 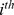 organ, and 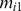 as the rate of lymphocyte migration from the 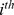 organ to the blood, and 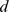 as the rate of removal of lymphocytes from circulation (due to death or migration to unsampled tissues), 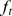 is the fraction of lymphocytes in SCLNs that return to the blood via the thoracic duct, and 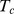 is the variable denoting whether lymphocytes from MLNs and SCLNs return to the blood (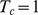) or are collected during thoracic duct cannulation (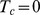). When fitting the first dataset (migration data, Figure 1A), we let 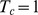 and when fitting the second dataset (cannulation data, Figure 1B) we let 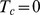. The total number of cells in the liver that includes dying cells, is 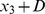. Note that 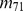 is the rate of migration of lymphocytes from Peyer's patches to the mesenteric LNs (eqn. (4)). In our model and experimental data 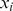 (or 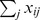) represents the total number of TDLs recovered from an organ which includes cells in the organ vasculature and parenchyma. Therefore, the estimated migration rates are for the total organ.

#### Thoracic duct cannulation

The rate of lymphocyte exit during cannulation experiments (Figure 1B, 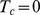 in eqn. (1)) is given by 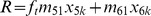 where 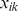 the percent of transferred lymphocytes in the last sub-compartment in SCLNs (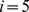) and MLNs (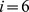) and 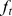 is the fraction of lymphocytes that exit the SCLNs into the blood via the thoracic duct. This structure is based on the known physiology of murine lymphatics where lymphocytes from MLNs and approximately half of lymphocytes from SCLNs enter the blood via the thoracic duct [68]. In our simulations we varied the number of sub-compartments 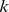 from 1 to 3; estimates of the average residence time of TDLs in LNs and PPs were dependent on the number of sub-compartments with a larger number of sub-compartments resulting in longer residence times. The model with two sub-compartments (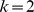) described the migration data (Figure 3) with best quality, and therefore, most results are shown for 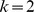. In order to explain cannulation data we assumed that during cannulation the rate of lymphocyte exit from LNs and PPs, 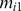, declines exponentially over time at a rate 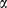 due to loss LN cellularity as has been suggested in previous studies [44], [45], [74].

#### Migration via antigen-stimulated lymph nodes

To describe migration of labeled TDLs via resting and antigen-stimulated popliteal LNs we use eqn. (3) and eqn. (5) and estimate the rate of lymphocyte entrance into LNs from the blood (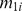) and the rate of lymphocyte exit from the LNs (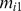). To simulate the dynamics of TDLs in the blood or other tissues we used parameter estimates shown in Table 1.

**Table 1:** Parameter estimates of the mathematical model and their 95% confidence intervals.

Organ	Rate of entrance from blood 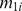 , 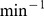	Percent cells going to organ from blood	Rate of exit from organ to blood 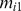 , 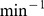	Residence time in organ, mins
Lung	1.83 (1.34–2.25)	78.2 (64.2–84.0)	2.17 (1.59–2.63)	0.46 (0.38–0.62)
Liver	0.41 (0.26–0.82)	17.4 (11.4–31.2)	1.14 (0.69–2.46)	0.88 (0.40–1.46)
Spleen	0.056 (0.051–0.061)	2.4 (1.8–3.0)	0.007 (0.0058–0.0087)	144 (115–173)
SCLNs	0.026 (0.024–0.028)	1.11 (0.85–1.36)	0.0034 (0.0029–0.004)	593 (498–693)
MLNs	0.0106 (0.0094–0.0119)	0.46 (0.34–0.56)	0.0034 (0.0029–0.004)	593 (498–693)
PPs	0.0053 (0.0044–0.0062)	0.23 (0.17–0.29)	0.0034 (0.0029–0.004)	593 (498–693)

We fit the basic mathematical model (eqn. (1) – (6)) to the the data on migration of thoracic duct lymphocytes in rats using nonlinear least squares. In total we estimate 12 model parameters. We list i) the rate of TDL entrance into a particular organ from the blood 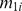 (second column), ii) the percent of cells leaving the blood into a particular organ (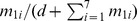, third column), iii) the rate of exit of TDLs from an organ to the blood 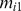 (fourth column), and iv) the average residence time of TDLs in the organ (fifth column). The rate of migration of TDLs from the blood to all organs, 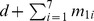, is 2.3 min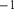 or the average residence time of cells in the blood is 26 sec. The average residence time of cells in a particular organ is calculated as 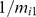 for blood, lung, liver, and spleen, and 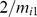 for SCLNs, MLNs, and PPs. Estimated fraction of cells migrating from the blood to the liver and dying is 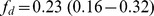 and the rate of cell migration from the blood to other organs in the body 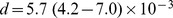 min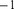. Exit rates from subcutaneous LNs, mesenteric LNs, and Peyer's patches (and, thus, residence times in these lymphoid tissues) are statistically similar as judged by the F-test for nested models (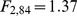, 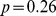), and therefore these rates were fitted as a single parameter. The average estimated residence time of TDLs in these secondary lymphoid tissues is 9.9 hours.

### Statistics

#### Basic analyses

For fitting of the mathematical model to migration or cannulation data we use nonlinear least squares [75]. Fits of our best models to percent data or to 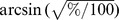-transformed data gave nearly identical parameter estimates (results not shown). For fitting of the model to both datasets (migration and cannulation data) we use a general likelihood approach [76], [77]. In this approach we assume that the distribution of errors for two datasets are normal but have different variances, 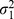 (migration data) and 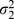 (cannulation data). For our model and the data, the general likelihood of observing the data given the model prediction on the percent of lymphocytes in a given organ 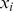 and the rate of lymphocyte exit during thoracic duct cannulation 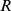 is
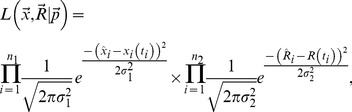
(7)where the products are taken for population of lymphocytes in all organs over all observation times, and 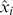 and 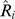 are experimental measurements, and 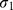 and 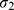 are the standard deviation of the errors in the migration and cannulation data, respectively, 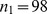 and 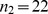 is the number of measurements, and 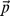 is the vector of model parameters to be estimated from the data. It is convenient to rewrite eqn. (7) in terms of log-likelihood 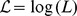:
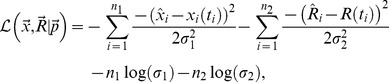
(8)where we omitted constant terms. Parameters were estimated by maximizing log-likelihood 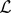. Model comparison was done using F-test for nested models [75] or Akaike Information Criterion, AIC [78]. Confidence intervals were estimated by bootstrapping the residuals with 1000 simulations [79].

## Results

### Rapid recirculation of lymphocytes between blood, lung, and liver

To study recirculation kinetics of thoracic duct lymphocytes (TDLs), Smith & Ford [65] adoptively transferred 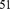
Cr-labeled TDLs and measured the percent of transferred lymphocytes in different organs of the recipient rats including the blood, lung, liver, spleen, skin-draining (subcutaneous) and gut-draining (mesenteric) lymph nodes (Figure 1). To estimate rates at which these TDLs migrate into different tissues of rats from the blood and the average residence times of TDLs in the tissues we developed a novel mathematical model. The model is based on a system of ordinary differential equations; the model takes into account recirculation of lymphocytes between lymphoid and nonlymphoid tissues via blood (Figure 2 and eqn. (1) – (6)). We fit this mathematical model to experimental data on the recirculation kinetics of TDLs in rats using nonlinear least squares. Despite its relative simplicity the model describes extremely well both the short and long term dynamics of TDLs (Figure 3A &B). In particular, the model accurately predicts a rapid drop in the percent of TDLs in the blood and concomitant increase in the percent of transferred TDLs in lung and liver within the first several minutes post transfer (Figure 3A). Over time, the percent of transferred cells residing in the blood, lung, and liver declines while transferred cells accumulate in the secondary lymphoid organs such as spleen, lymph nodes, and Peyer's patches (Figure 3B). The early decline in the fraction of labeled TDLs was similar in blood, lung, and liver suggesting a rapid recirculation of lymphocytes between these compartments.

In the first 30 minutes of the experiment the spleen accumulated slightly more transferred lymphocytes than all lymph nodes and Peyer's patches together (55 vs 45%). The percent of TDLs in the spleen started to decline in about 1.5 hours after cell transfer suggesting that residence time of TDLs in the spleen is relatively short. By the end of the 24 hour experiment the majority of the transferred cells (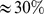) were found in subcutaneous lymph nodes (SCLNs). The model also captures well the dynamics of the total percent of TDLs in animals after transfer (Figure 2 in Text S1). Nearly 90% of transferred lymphocytes were recovered from the seven murine organs at 24 hours post-transfer (Figure 2 in Text S1).

Using our mathematical model we estimated that TDLs exit the blood very quickly at a rate 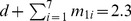 min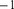, which corresponds to the average residence time of TDLs in the blood of 26 seconds (Table 1). The majority of lymphocytes leaving the blood enter the lung (78%) and liver (17%) with only 5% of all lymphocytes in the blood migrating to the secondary lymphoid organs. Even though there is a rapid migration of lymphocytes from the blood vessels into lung and liver there is no accumulation of lymphocytes in the latter tissues. According to our model TDLs that entered the lung and liver return back to the blood after, on average, only 28 and 53 seconds, respectively (Table 1). Such rapid migration via these tissues suggests that TDLs found in the lung and liver are most likely located in the vasculature of these organs (capillaries of the lung and sinusoids of the liver). These data in combination with our mathematical model suggest that migration of lymphocytes via lung and liver vasculature in normal, noninflammatory conditions is very rapid. The combined rapid exit of TDLs from the blood into vasculature and rapid return back to the blood results in a relatively long average residence time of TDLs in the blood as has been observed in this and many other studies [25], [40], [65], [80]–[83]. Thus, our modeling reveals a highly dynamic nature of recirculation of thoracic duct lymphocytes between blood, lung capillaries, and liver sinusoids in rats.

### Migration kinetics of lymphocytes via spleen and major lymph nodes

While there is a rapid recirculation of TDLs between the blood, lung, and liver, the percent of TDLs found in the blood declines slowly over time (Figure 3B). The decline cannot be due to cell death because the total percent of TDLs recovered from all major tissues declines very little (Figure 2 in Text S1). This decline in the percent of TDLs found in the blood is due to lymphocyte migration to secondary lymphoid organs such as the spleen, lymph nodes, and Peyer's patches. While migration into secondary lymphoid organs is slow and only 5% of lymphocytes found in the blood migrate into spleen, LNs, and PPs (Table 1), because of long average residence times of TDLs in these tissues, spleen, LNs, and PPs act as sinks for lymphocytes: whichever lymphocyte enters these tissues it does not leave for an extended period of time. We estimate that the average residence time of TDLs in the spleen is 2.4 hours while it takes on average 10 hours for TDLs to leave lymph nodes or Peyer's patches (Table 1). Interestingly, the average residence time of TDLs in different lymph nodes and Peyer's patches was found to be the same (Table 1). Allowing for different rates of lymphocyte egress from SCLNs, MLNs, and PPs did not improve the quality of the model fit to data (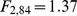, 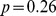). The result of similar average residence times of lymphocytes in LNs and PPs was strongly dependent on the assumption that lymphocytes leaving Peyer's patches migrate to the mesenteric lymph nodes. If we let lymphocytes from PPs migrate directly to the blood, we found different residence times of lymphocytes in SCLNs, MLNs, and PPs (results not shown).

### Predicting lymphocyte exit from LNs using thoracic duct cannulation data

**Figure 4:**
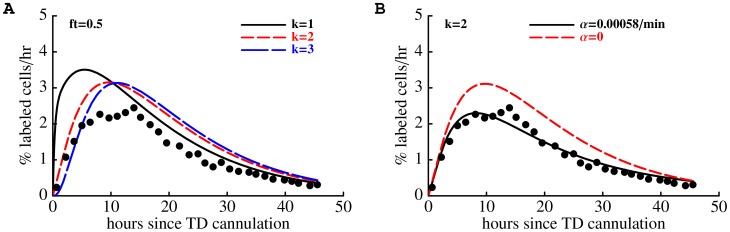
Increase in the average residence time of lymphocytes in lymph nodes with time since cannulation is needed to explain the kinetics of labeled lymphocyte exit during thoracic duct cannulation. 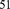
Cr-labeled TDLs were passaged via an intermediate host and then transferred into final recipient rats. Recipients were cannulated via the thoracic duct (Figure 1B) and the rate of exit of labeled TDLs into the thoracic duct per hour was measured [65]. The data are shown by markers (points). In panel A we show that for the parameter estimates from migration experiments (Table 1) the models with different number of subcompartments in LNs (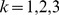) fail to describe experimental data when 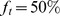 of lymphocytes exiting SCLNs migrate to the blood via the thoracic duct. To explain the data, we let the rate of lymphocyte exit from the LNs to decline exponentially with the time since cannulation, 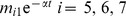 (panel B). We fit the data on the output rate of labeled cells into the thoracic duct using the mathematical model (eqn. (1) – (6)). We fix all model parameters to values shown in parameters for 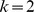 and fit only parameters 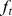 and 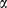. The best description of the data was found when 1) the fraction 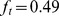 of lymphocytes in SCLNs enter the blood via the thoracic (left lymphatic) duct, and 2) the rate of lymphocyte migration via lymph nodes and Peyer's patches declines with time since cannulation at a rate 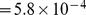 min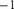 (solid line in panel B). The model fails to predict thoracic duct output data if residence times of lymphocytes in LNs is unaffected by cannulation (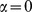, large dashing lines in panels A and B).

Our model describes well the dynamics of labeled lymphocytes in the whole rat within the first 24 hours after cell transfer (Figure 3). We next sought to investigate whether our model is also able to predict the kinetics of exit of transferred TDLs via the thoracic duct in an additional set of experiments [65]. In these experiments 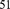
Cr-labeled TDLs were passaged via an intermediate host and then were transferred into final recipient rats. The recipients were then cannulated via the thoracic duct (Figure 1B) and the rate of labeled lymphocyte exit per unit of time was recorded (Figure 4).

The rate of lymphocyte exit into the blood via the thoracic duct depends strongly on the fraction of lymphocytes exiting into blood via left (thoracic) and right lymphatic ducts and on the time lymphocytes spend in lymph nodes. Thoracic duct collects lymphocytes exiting mesenteric lymph nodes and lymphocytes exiting subcutaneous lymph nodes on the left side of the body [68], [84]. Assuming that around 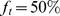 of lymphocytes from SCLNs enter the blood via the thoracic duct, the model prediction on thoracic duct output rate of labeled cells depends strongly on the distribution of residence times of lymphocytes in LNs (Figure 4A). The model in which residence times are exponentially distributed (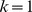) does not predict the shape nor the total number of lymphocytes exiting LNs into the thoracic duct. Models with gamma-distributed residence times (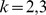) explain well the general shape of the cannulation data such as initial increase, peak, and long tail but also fail to explain the total number of cells recovered during cannulation which is given by the area under the data (Figure 4A, dashed lines). In particular, in the data only 55% of lymphocytes exit the lymph nodes into the thoracic duct in 45 hours (Figure 4) while the model at 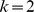 predicts that 78% of lymphocytes should exit lymph nodes by that time. This result suggests that cannulation influences the kinetics of lymphocyte migration via lymph nodes. We tested a previously suggested mechanism that reduction in cellularity of lymph nodes leads to increased retention of lymphocytes in the nodes [44], [45], [74], [85]. Specifically, previous work suggested that it takes longer for lymphocytes to pass from blood to efferent lymph in irradiated or nude recipients which have reduced lymphocyte numbers in spleen and LNs [44], [74], [85]. Furthermore, in a recent study where lymphocyte entrance into lymph nodes was blocked with anti-CD62L antibodies, the rate of lymphocyte loss from LNs was predicted to decrease with time since blockage ([45], see also Figure 3 in Text S1).

In our model, cannulation leads to an exponential decrease in the total number of lymphocytes in all tissues (results not shown), and therefore, we let the rate of lymphocyte migration via the lymph nodes and Peyer's patches to decline exponentially over time during cannulation due to loss of LN cellularity. Under the assumption that the exit rate from LNs and PPs declines exponentially with the time since cannulation, the model with 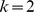 (Figure 4B) or 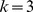 (not shown) matches well the kinetics of migration of labeled TDLs via the thoracic duct predicting both the shape and total magnitude of the cannulation data. The model suggests that in 24 hours of thoracic duct cannulation, the average residence times of lymphocytes in the LNs increases 2 fold, from 10 to 23 hours, and this in part explains long time that would be required to collect all transferred lymphocytes via the thoracic duct. Importantly, the model in which residence times of lymphocytes in LNs are exponentially distributed failed to explain the cannulation data even at 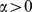 (results not shown). An alternative mechanism in which cannulation leads to exponentially increasing cell death rate also failed to explain the cannulation data (results not shown).

### Increase in the size of antigen-stimulated LN is due to increased entrance rate of lymphocytes and not decreased exit rate

**Figure 5:**
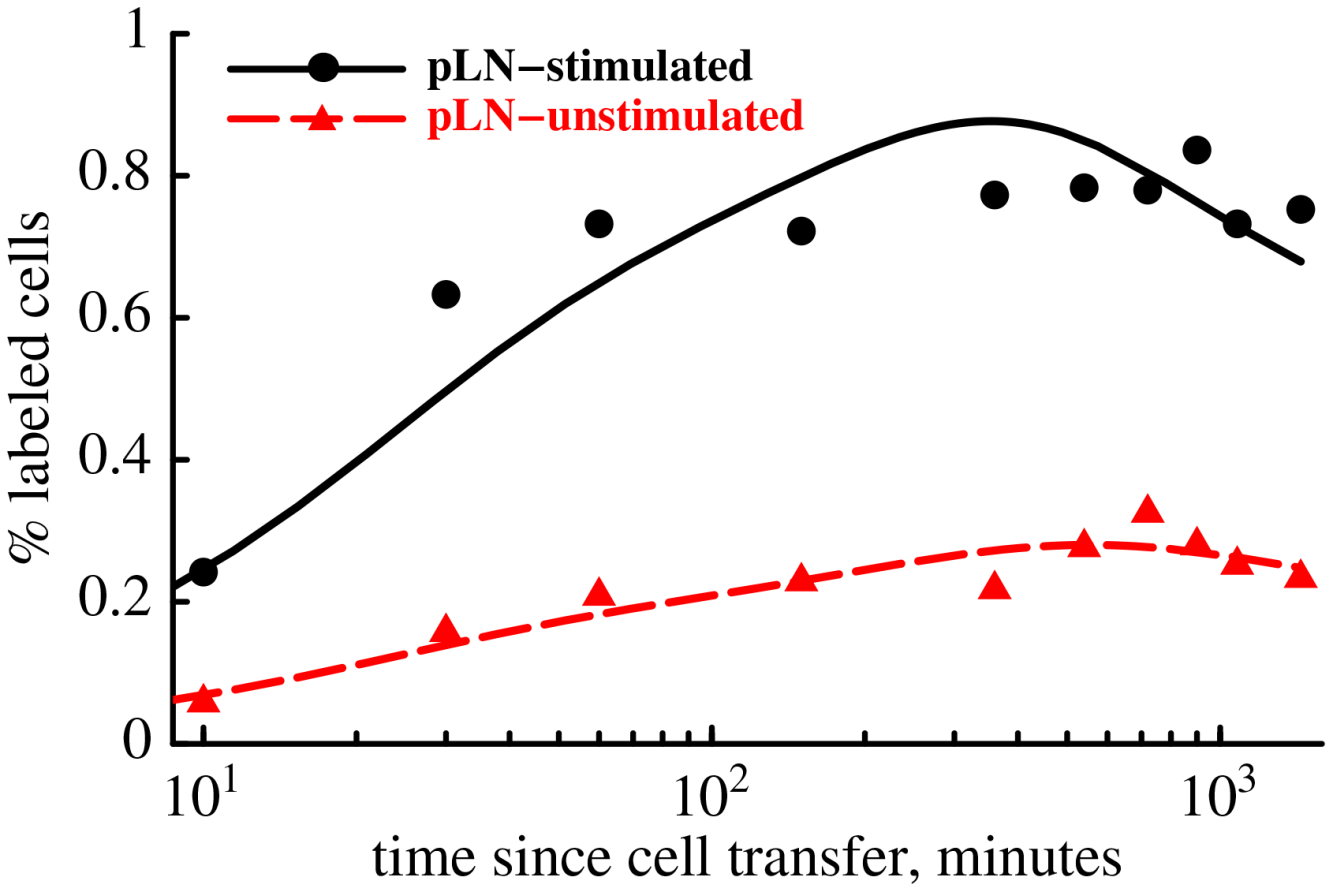
Antigen-stimulated popliteal lymph nodes (pLN) accumulate higher numbers of 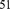
Cr-labeled TDLs because of increased entrance rate of TDLs into the LN. We plot the percent of labeled TDLs found in the antigen-stimulated (dots) and resting (triangles) popliteal LNs. Model fits of the data are shown by lines. We found that the entrance rate into stimulated and unstimulated pLNs is 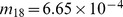 min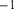 and 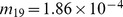 min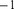, respectively. Exit rate of lymphocytes from antigen-stimulated and unstimulated pLNs are 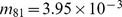 min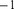 and 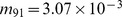 min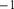 (two sub-compartments in the LNs, 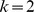). To describe the dynamics of TDLs in the blood and other organs we used parameters given in Table 1. Our results suggest that antigenic stimulation of the lymph node with sheep erythrocytes increases the rate of entrance of TDLs into the LN almost 4 fold without a significant change in the lymphocyte exit rate (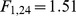, 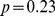).

It is well established that antigen-stimulated lymph nodes increase in size [86]–[89]. However, the reasons for this size increase have been debated [88]. Some studies have argued that a reduced rate of exit of cells from lymph nodes [69], [86], [87], [90] while others suggested that an increased entry of lymphocytes into lymph nodes [89], [91]–[93] leads to increase in size of antigen-stimulated lymph nodes. Because it has been difficult to simultaneously measure the rate of lymphocyte entrance into and the rate of lymphocyte exit from a given lymph node, it remains unresolved whether both processes (reduced exit rate and increased entrance rate) contribute to the LN enlargement. We address this question by analyzing an additional set of data from experiments performed by Smith & Ford [65]. The authors stimulated popliteal LN (pLN) of rats with sheep erythrocytes 3 days prior to transfer of 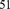
Cr-labeled thoracic duct lymphocytes, and another pLN was left unstimulated. More labeled lymphocytes accumulated in the antigen-stimulated pLN than in the control pLN (Figure 5). By fitting the mathematical model to these data we find that a larger percent of labeled lymphocytes in the antigen-stimulated pLN was due to almost 4 fold increase in the rate of entrance of lymphocytes from the blood into the LN (Figure 5). The rate of lymphocyte egress from stimulated and unstimulated pLNs 3 days after antigen delivery was the same (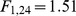, 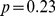). Importantly, the model did not match well the data on lymphocyte migration via the antigen-stimulated lymph node suggesting that rates of lymphocyte entry into and exit from such lymph nodes may be time-dependent. Indeed, allowing for the entry and/or exit rates to change exponentially with time since TDL transfer improved the model fit to these data (Figure 4 and Table 1 in Text S1). However, the predicted changes in the exit/entrance rates with time appeared to be biologically unrealistic (see Figure 4 in Text S1). Further work is needed to understand whether and how rates of lymphocyte entrance into and exit from antigen-stimulated LNs change over the course of infection.

## Discussion

Our study for the first time provides quantitative estimates of migration rates of thoracic duct lymphocytes into and the average residence time of TDLs in major murine organs in one modeling framework. Several results are particularly interesting. We find very rapid recirculation of TDLs between major blood vessels and vasculature of the lung and liver, which can also be readily seen in the data. In 1 minute the percent of TDLs in the blood drops from 100% to 40% which already implies that half-life time of lymphocytes in the major blood vessels is less than one minute (Figure 3A). In the same time period, the lung receives 35% and liver 17% of transferred TDLs. This suggests that migration of TDLs into the lung and liver is extremely rapid. The percent of TDLs in these two organs does not accumulate dramatically over time suggesting rapid exit of lymphocytes from these organs into the circulation. Relatively rapid migration of thoracic duct and blood lymphocytes via the vasculature of the lung was also noticed in previous studies in rats or pigs [40], [82]. Previous studies on migration of neutrophils found that although the majority of neutrophils are delayed during their passage via the lung vasculature, the average transit time of neutrophils via the vasculature in humans is only around 3 minutes [54], [94]. This is well consistent with our proposed estimate for the average residence time of TDLs in the murine lung vasculature (Table 1).

In our experimental data measurements of transferred cells in the lung tissues included both cells in vasculature (capillaries) and cells in parenchyma [65]. Consequently, our model describes TDL migration via the lung as migration via a one compartment organ. Predicted by the model rapid migration of TDLs via the lung suggests that lymphocytes pass the lung via the lung capillaries. However, it is possible that some lymphocytes may gain access to lung parenchyma and may reside in the tissue for substantial time before migrating to the lung-draining lymph nodes via afferent lymphatics. Indeed, Caucheteux *et al*. [64] recently observed that some naive CD4 T cells may be recovered from lung parenchyma after adoptive transfer into naive mice. In their experiments, Caucheteux *et al*. [64] transferred naive P25 TCR transgenic CD4 T cells, specific to pigeon cytochrome C protein, into naive recipient and 24 hours later, treated the recipient mice with the drug FTY720. FTY720 binds to S1P receptor on lymphocytes, thus preventing their egress from lymph nodes [18], [95], [96]. Following FTY720 treatment, Caucheteux *et al*. [64] observed a rapid decline in the density of donor T cells in the blood and a much delayed decline of donor cells in the lung parenchyma suggesting that donor T cells in the blood and the lung parenchyma are not in equilibrium. We extended our mathematical model to include migration of TDLs from the lung vasculature to parenchyma and then to lung-draining medistinal lymph nodes (SCLNs in our model). If lymphocytes reside in the lung parenchyma for over 10 hours [64], our model can well describe the dynamics of lymphocytes via the whole lung (vasculature and parenchyma) if a small percent (e.g., 0.01%) of lymphocytes exiting vasculature migrate to the lung parenchyma (results not shown). Taken together, our results and work of Caucheteux *et al*. [64] suggest that the bulk of naive T cells travel rapidly via the lung vasculature while a small fraction of lymphocytes may enter lung parenchyma and may remain the tissue for an extended period of time. Detailed understanding of lymphocyte migration via lung vasculature and parenchyma can be obtained when kinetic data on the lymphocyte distribution between these compartments become available.

By considering migration of lymphocytes via lung/liver or secondary lymphoid organs (SLO such as spleen, LNs, and PPs) as a Bernoulli trial we can estimate the average number of times a lymphocyte in the blood will pass via lung or liver vasculature before migrating into the SLO. Given that the probability of migrating via vasculature is 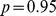 and via SLO is 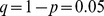 (Table 1), the average number of “failures” (travel via lung or liver) before first success (travel via SLO) is 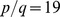. Such a large number of passes via lung or liver before lymphocyte enters a lymph node or spleen may explain why during some pulmonary infections many lymphocytes can be recovered from the lung. If inflammation in the lung increases the time that lymphocytes require to pass via the lung vasculature, many lymphocytes are expected to accumulate in the lung without an often postulated specific antigen-dependent recruitment of effector T cells to the lung vasculature (Table 2).

**Table 2:** Predicted steady state distribution of the percent of TDLs in a 1) control animal, 2) following increased in the average residence time of lymphocytes in the lung (20 fold, from 26 seconds to 9 minutes) due to, for example, inflammation in the lung; 3) following a decreased entrance rate of TDLs into lymph nodes and Peyer's patches (20 fold, e.g., by using anti CD62L antibody); 4) following a decreased exit rate of TDLs from lymph nodes and Peyer's patches (5 fold, e.g., using FTY720).

Organ	Control	Increased residence in lung (inflammation)	Blocked LN entrance (anti CD62L Ab)	Blocked LN exit (FTY720)
Blood	2.6 (2.2–3.2)	1.8 (1.6–2.1)	8.6 (7.6–9.9)	0.7 (0.6–0.8)
Lung	2.2 (1.9–2.7)	**31.1 (27.6–35.5)**	7.3 (6.4–8.4)	0.6 (0.5–0.7)
Liver	0.9 (0.8–1.2)	0.7 (0.5–0.8)	3.1 (2.6–3.8)	0.2 (0.2–0.3)
Spleen	20.9 (19.5–22.1)	14.7 (13.6–15.6)	**68.9 (66.3–70.8)**	5.3 (4.9–5.7)
SCLNs	**40.3 (38.8–41.4)**	28.4 (26.18–30.1)	6.6 (6.18–7.2)	**51.2 (49.58–52.8)**
MLNs	24.8 (23.58–26.1)	17.5 (15.8–19.1)	4.1 (3.7–4.6)	31.5 (30.2–32.8)
PPs	8.3 (7.2–9.3)	5.8 (5.0–6.7)	1.4 (1.2–1.6)	10.5 (9.3–11.8)

We let the model with parameters estimated from experimental data (Table 1) to reach a steady state (control), and then various treatments are applied and the predicted steady states are shown. The highest predicted percent of TDLs in different scenarios is highlighted in bold. Confidence intervals (95%) shown in brackets are estimated using bootstrapped values for migration rates from Table 1. In calculation we assume that after 24 hours there is no migration of TDLs to other organs (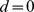), and the percents shown in the table have been normalized to have the total of 100% of TDLs at the steady state.

It is useful to compare the estimated residence times of lymphocytes in vasculature of the lung and liver with the kinetics of recirculation of the blood. Given that the total volume of blood in 170 gram rats is about 11 ml [97], the volume of the heart is about 1 mL, heart beat rate is 300 per min, and that about 30% of the heart volume is pumped by heart per beat, we find that in 1 minute, rat heart will pump 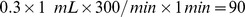 mL of blood. This suggests the average turnover rate of the blood is approximately 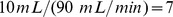 sec which is 5 to 10 fold smaller than the time it takes for lymphocytes to travel via lung and liver vasculature, respectively. Why it takes longer for lymphocytes to migrate via liver sinusoids than via lung capillaries remains to be determined.

**Figure 6:**
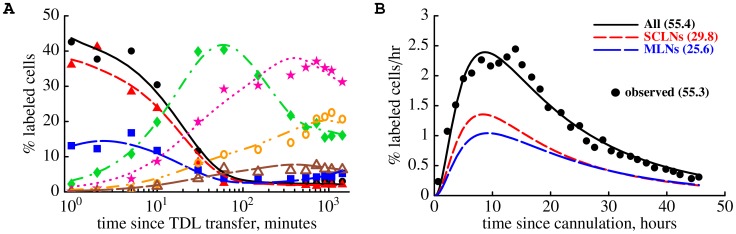
The mathematical model accurately describes the kinetics of lymphocyte migration via major lymphoid organs (panel A) and kinetics of labeled lymphocytes exit during the thoracic duct cannulation (panel B). We fit the basic mathematical model (eqn. (1) – (6)) simultaneously to the data on lymphocyte migration (Figure 3) and lymphocyte output via the thoracic duct (Figure 4) using generalized likelihood method assuming 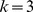 subcompartments in LNs and PPs (see Materials and Methods). In panel A, symbol and line labeling is similar to that of Figure 3. The model predicts that the 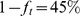 of lymphocytes exiting SCLNs migrate to the blood via the right lymphatic duct and thus are not sampled during the thoracic duct cannulation. Numbers in parentheses in panel B indicate the percent of cells exiting into blood from different LNs in 45 hours as predicted by the model (lines) or as observed in the data (dots). In the data, 55% of transferred TDLs were collected during 45 hours of the thoracic duct cannulation. The model also predicts the contribution of lymphocytes exiting SCLNs (short dashed line in panel B) and MLNs (long dashed line in panel B) via the thoracic duct. To explain the data, the rate of egress of lymphocytes from LNs and PPs declines exponentially with time during cannulation at an estimated rate 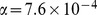 min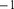. Other parameters for the migration kinetics of TDLs are nearly identical to those given in Table 1. We fixed 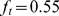 in fits of these data. Estimated standard errors are 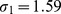 and 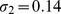 (see eqn. (8)).

By fitting our mathematical model to two sets of data simultaneously (migration data and cannulation data) we confirmed our estimates of the average residence times of TDLs in the spleen and lymph nodes (Figure 6). While our estimates of the average residence time in the spleen, lymph nodes, and Peyer's patches provide a consistent ranking of lymphoid tissues in terms of their lymphocyte retention potential (e.g., lymphocytes stay longer in the LNs than in the spleen), our estimates for lymphocyte residence times in LNs are smaller than previously suggested values [25], [35], [98] including estimates obtained in recent studies utilizing mathematical modeling [33], [45], [46]. Interestingly, our prediction on the average residence time of TDLs in the Peyer's patches is similar to the reported half-life time of B cells in mouse PPs [47]. However, as we have discussed previously, it is not straightforward to calculate the average residence time from the half-life times in the absence of information on the underlying probably distribution of residence times [99].

It remains unclear why our estimates of the average residence time of lymphocytes in LNs are shorter than recently published values obtained using adoptive transfer of naive or memory T cells in mice [45], [46]. There could be several explanations.

First, it could be the type of lymphocytes. Lymphocytes used in our experimental data have been passaged through an intermediate host from blood to thoracic duct lymph prior to final transfer (see Materials and Methods). It has been argued that long handling of lymphocytes *in vitro* and isolation of lymphocytes from lymphoid tissues such as spleen and LNs can drastically influence their migration kinetics [65], [66], [100]. In particular, thoracic duct lymphocytes transferred to intermediate hosts and then isolated from lymph nodes have drastically reduced ability to migrate to lymph nodes of new recipients within first 30 min post transfer [100]. It is interesting to note that TDLs used in our experimental data migrate much quicker to the spleen as compared to spleen-derived lymphocytes (splenocytes). Previous studies estimated that splenocytes injected intravenously into recipient mice migrate to the spleen at a rate 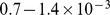 min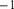 [101], [102] while in our data, TDLs migrate from the blood to the spleen at a rate 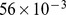 min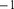 (Table 1). Such a large difference is unlikely to arise due to different types of lymphocytes used in experiments - spenocytes are mostly B cells while TDLs are mostly T cells [100]. The slow predicted rate of splenocyte migration to the spleen in previous experiments [101], [102] could be due to the assumption that all transferred splenocytes (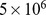) are capable of migrating to and suriving in the spleen. If only 10% of transferred cells have this ability, the difference in estimated migration rates between splenocytes and TDLs would be only 4 fold.

Second, it could be due to assumptions made in the previous mathematical models used to analyze the data [45], [46]. Both studies used adoptive transfer of lymphocytes into syngenic hosts and added antibodies after the cell transfer to block entry of donor lymphocytes into lymph nodes. In the mathematical models the authors assumed that the residence times of lymphocytes in LNs are exponentially distributed and that efficacy of antibodies at blocking entry of cells into the nodes is 100%. Changing both of these assumptions may influence the observed decline of the donor cells in the LNs (Figure 5 in Text S1 and result not shown). In particular, if antibody blocking efficacy is not 100% then the rate of decline of the density of donor cells will underestimate the true lymphocyte egress rate from LNs (Figure 5 in Text S1), and thus will provide a higher estimate of the average residence time.

Third and finally, differences in estimates could arise due to different experimental techniques used in different studies. In particular, it is not known if death could be contributing to the loss of cells from LNs during the antibody blockade experiments. In these experiments lymphocytes exiting LNs were never counted back in other places to make sure that cell loss due to death does not play an important role in these experiments [45], [46]. Inclusion of cell death in the models will likely increase the estimates of the average residence times of lymphocytes in LNs.

Our estimates of TDL residence times in LNs are also shorter than recently calculated residence times for blood lymphocytes migrating via individual lymph nodes in sheep [33]. There could be species-specific differences in residence times between rats and sheep. Importantly, however, the mathematical model of Thomas *et al*. [33] did not incorporate a continuous input of lymphocytes from the blood into the lymph node and that assumption likely led to overestimation of the lymphocyte residence time in the nodes (McDaniel and Ganusov, unpublished work).

Our mathematical model correctly predicts the exit rate of labeled lymphocytes into the circulation via the thoracic duct (Figure 4) when 1) 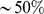 of lymphocytes in the subcutaneous LNs enter the blood from lymph nodes via the thoracic (left lymphatic) duct, 2) distribution of residence times of lymphocytes in lymph nodes follows a right-tailed gamma distribution (with the shape parameter 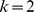 or 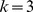), and 3) the rate of lymphocyte exit from lymph nodes declines exponentially with the time since cannulation (Figure 4). Assuming that lymphocyte residence times in lymph nodes follow an exponential distribution as has been recently suggested [46], we were unable to explain the kinetics of exit of transferred lymphocytes during thoracic duct cannulation. This model predicted a much earlier peak in the rate of lymphocyte exit into the thoracic duct than that observed in the data (Figure 4A). Analysis of the data for migration of lymphocytes via individual lymph nodes [28] also suggested that the residence times in lymph nodes cannot be given by the exponential distribution because there is always a substantial delay before the majority of labeled lymphocytes is detected in efferent lymph (McDaniel and Ganusov, unpublished observations). The conclusion that residence times of naive T lymphocytes in lymph nodes are exponentially distributed came from the observation that blocking entrance of lymphocytes into LNs leads to an exponential decline in the number of adoptively transferred T cells recovered in LNs. Using parameter estimates in our model (Table 1) we simulated these experiments assuming different levels of the efficacy of blocking lymphocyte entry into LNs (Figure 5 in Text S1). The results of the simulations suggest that even if the distribution of residence times in LNs is not exponential, the number of transferred cells after blockade may still decline exponentially (Figure 5 in Text S1). Therefore, the observation of exponential-like behavior should not be taken as evidence that the underlying probability distribution of residence times is exponential [99], [103].

In our mathematical model we assumed that lymphocytes exiting LNs appear instantaneously in the blood. This is clearly a simplification since lymphocytes must travel with lymph via lymphatic ducts before they enter circulation (Figure 1 in Text S1). We were unable to find direct estimates of the time of lymphocyte migration via right lymphatic or thoracic ducts in rats. However, knowledge of the basic dimensions of the ducts and the rate at which lymph can be collected during thoracic duct cannulation can be used to make a reasonable estimate. The length of the thoracic duct varies with the weight of the rat between 3 and 7 cm [104, and unpublished observations of Michael D. Karlstad UT Medical Center]; the largest duct we found was 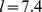 cm (M.D. Karlstad). The internal diameter of the thoracic duct varies from 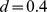 to 1 mm [105], [106], and this makes the internal volume of the thoracic duct to be 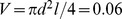 cm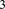 for 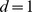 mm. Finally, the rate of lymph flow during thoracic duct cannulation in rats varies between 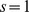 to 10 ml per hour [107], [108]. Assuming that lymphocytes in the thoracic duct move with the lymph, the average residence time of lymphocytes in the thoracic duct is then 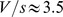 min for 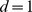 ml/h. These calculations suggest that migration via the thoracic duct (and likely via the right lymphatic duct) is extremely rapid as compared to migration via lymph nodes, and this supports our assumption that TDLs exiting LNs enter the circulation nearly instantaneously.

Using our mathematical model we can predict the long term distribution of labeled TDLs in different murine organs (Table 2). We predict that at the steady state 2.6% of lymphocytes will be found in the blood, 21% in the spleen, and 65% in the LNs (Table 2:column 2). This is in perfect agreement with actual observations at 24 hours post transfer [65 Figure 3]. The model also can be used to predict how changes in the rates of migration of lymphocytes to lymphoid or nonlymphoid tissues (or changes in residence time) may influence the distribution of lymphocytes in the body (columns 3–5 in Table 2 and Figures 6–8 in Text S1). The analysis demonstrates that increasing the average residence time of lymphocytes in the lung to only 9 minutes will result in predominant accumulation of TDLs in the lung vasculature. This result suggests that accumulation of lymphocytes in the lung in some (e.g., inflammatory) conditions may simply result from a slightly longer residence time of lymphocytes in the lung capillaries [28], [45], [57], [58], [109]. Indeed, recent work suggested that activated CD8 T cells may accumulate in the lung before traveling to the site of infection [109]. Such accumulation can be simply due to increased residence time in the vasculature of the lung and not because of directed migration of activated lymphocytes to the lung [56].

Similarly, blocking entrance of lymphocytes into LNs or egress of lymphocytes from LNs will dramatically change composition of lymphocytes in lymph nodes and circulation (Table 2:columns 4 and 5). Our simulations suggest that blocking entrance of lymphocytes into LNs has the major influence on the number of lymphocytes in nonlymphoid organs such as lung and liver and blocking exit of lymphocytes from LNs leads to only a moderate increase in lymphocyte numbers in LNs. This is in good qualitative agreement with experimental observations [110].

There are a number of potential limitations with the experimental data of Smith & Ford [65] and the assumptions made in our mathematical model. First, TDLs in these experiments were labeled with a radioactive label and after cell transfer, radioactivity from a given tissue was used as an indication of the percent of the initially transferred TDLs. Previous studies have established that in most cases labeling with 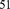
Cr does not induce excessive cell mortality and does not strongly influence their migration patterns [6], . Furthermore, dying cells tend to accumulate in the liver and kidney [6] and this has been often used to judge the quality of preparation of labeled cells. Measuring radioactivity from a relatively intact tissue has its advantages in comparison with flow cytometry from tissue-derived single cell suspensions since the former method does not involve any cell loss. Recently it has been shown that preparation of single cell suspensions from peripheral tissues may be associated with severe (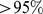) loss of memory T lymphocytes [112].

Second, in experimental data of Smith & Ford [65], the precise location of transferred lymphocytes within a given tissue is not known. Estimation of the rates of lymphocyte migration to parenchymal tissues of the lung and liver will require additional levels of experimental data, e.g., measurement of cells in the vasculature vs. parenchymal tissues [7], [13], [22], [56].

Third, in our model we assumed that lymphocytes enter LNs and PPs via the blood. It is well known that lymphocytes can leave the circulation into tissues and then accumulate in lymph nodes via afferent lymphatics. It appears, however, that to describe these experimental data there was no need to make this extra assumption although we cannot exclude that some labeled TDLs did enter lymph nodes and Peyer's patches via the afferent lymph [72]. How lymphocyte migration to lymph nodes via afferent lymph may influence parameter estimates for lymphocyte recirculation kinetics remains to be investigated.

Fourth, our mathematical modeling only provides estimates of migration of lymphocytes via a collection of lymph nodes or Peyer's patches and not transit times via individual nodes. This is because accumulation and loss of labeled cells in individual lymph nodes was not available in the original publication [65]. Experimental data that track migration of labeled cells in individual lymph nodes will be important to determine if lymphocytes migrate at different rates via lymph nodes that are organized in chains [68].

Finally, cellular composition of the thoracic duct lymphocytes used by Smith & Ford [65] is not entirely clear. Previous work established that in naive animals, TDLs consist of mainly naive B and T cells [40], and in rats, T to B cell ratio in TDL suspensions are 70%/30% [100]. Because naive T cells appear to migrate faster from the blood to lymph as compared to naive B cells [40], and CD4 T cells may have a shorter residence time in LNs than CD8 T cells [46], a passage via an intermediate host may have led to enrichment of naive CD4 T cells in the population of TDLs used in migration experiments (Figure 3). Indeed, it was noted that passaging of TDLs via an intermediate host increases the relative abundance of T cells in the thoracic duct lymph from 70% to 80–85% [100]. Thus, our results are most likely applicable to migration kinetics of naive T cells (and possibly naive CD4 T cells).

Given our prediction that the distribution of residence times of lymphocytes in lymph nodes is unlikely to be exponential, different approaches could be used to model lymphocyte migration. We opted for a simpler approach using ordinary differential equations with several sub-compartments for lymph nodes. We should stress, however, that such subcompartments need not represent actual physiological entities. Rather they should be viewed as a mathematical method to allow for non-exponentially distributed residence times of lymphocytes in tissues. An alternative approach may involve the use of standard structured population models which, however, often involve more parameters and are more difficult to fit to experimental data [103], [113].

Our model predictions on the hierarchy of lymphocyte recirculation kinetics in the whole organism are highly robust. We have fitted several alternative models to the migration data and all of these fits resulted in quantitatively similar parameter estimates (results not shown). We believe that this result comes from the strong constrains put in our model by the physiology of lymphocyte recirculation in rats and by experimental data. Yet, the average residence time of lymphocytes in the LNs and PPs did depend on the number of sub-compartments in these lymphoid tissues which is defined by the shape parameter of the gamma distribution (results not shown). Development of more realistic models of lymphocyte migration via lymph nodes should improve our estimates of the average residence time in these secondary lymphoid tissues.

Our analysis illustrates the power of mathematical modeling in quantifying lymphocyte migration kinetics, raises a number of important questions, and opens up avenues for future research. It is unclear if our results hold for lymphocytes isolated from other tissues such as blood, bone marrow, spleen, lung, or different LNs. Previous work suggested minimal differences in accumulation of lymphocytes isolated from the thoracic duct, LNs, or spleen in different lymphoid tissues at 24 hours after cell transfer [100] but more research is needed. Furthermore, quantitative estimates of the rates of migration of different types of lymphocytes (B vs. T cells) or different subsets of lymphocytes (naive/memory/effector) to spleen or nonlymphoid organs such as gut or skin are missing. There is now evidence for memory T cells residing in many nonlymphoid tissues [9], [10], [12]–[15] including resident T cells in LNs [114]. There is yet no direct evidence for spleen-resident lymphocytes. It also remains unclear whether tissue-resident T cells are true residents or they simply have a relatively long residence time in a given tissue. We now know that other cells such as dendritic cells and CD4 T cells are able to control entrance of lymphocytes into LNs [93], [115]. It remains to be determined whether lymphocyte entrance into and more importantly exit from nonlymphoid tissues is similarly controlled by immune cells [22]. Our study illustrates that transfer lymphocytes from syngenic animals and recording the percent/number of lymphocytes in multiple organs/tissues at different times after transfer is a powerful method to study lymphocyte migration patterns especially when it is combined with mathematical modeling.

## Supporting Information

Dataset S1 Experimental data from Smith and Ford (1983) [65] on migration of thoracic duct lymphocytes to multiple tissues of rats (migration data).
(CSV)

Dataset S2 Experimental data from Smith and Ford (1983) [65] on the rate of exit of thoracic duct lymphocytes into the thoracic duct (cannulation data).
(CSV)

Dataset S3 Experimental data from Smith and Ford (1983) [65] on accumulation and loss of thoracic duct lymphocytes in resting and antigen-stimulated popliteal lymph nodes of rats.
(CSV)

Text S1 Additional results of the analysis.
(PDF)
