# Supplementary material for: Mathematical Modeling Reveals Kinetics of Lymphocyte Recirculation in the Whole Organism
Source: PLoS Comput Biol. 2014 May 15;10(5):e1003586. doi: 10.1371/journal.pcbi.1003586 (PMC4022467; doi:10.1371/journal.pcbi.1003586)
Supplement: Text S1 — Additional results of the analysis. (PDF) [file pcbi.1003586.s004.pdf]

Text S1

LN type	Initial entrance rate m_{1i}, 10^{-4} min^{-1}	Initial exit rate m_{i1}, 10^{-3} min^{-1}	Change in entrance rate γ_1, 10^{-3} min^{-1}	Change in exit rate γ_2, 10^{-3} min^{-1}
resting pLN	1.86 (1.38-2.58)	3.07 (1.82-4.62)	0*	0*
Ag-stimulated pLN	6.65 (6.10-7.42)	3.95 (3.49-4.49)	0*	0*
Ag-stimulated pLN	7.03	5.08	0.32	0*
Ag-stimulated pLN	8.8	8.07	-9.38	-9.38

Table 1: Estimates of the rates of entry into and exit from antigen-stimulated and resting popliteal lymph nodes. We fix parameters for the lymphocyte recirculation kinetics (given in Table 1 in the Main text) and estimate the rate of lymphocyte migration into resting pLN and pLN stimulated 3 days previously with sheep red blood cells. When fitting the data on the dynamics of lymphocytes in Ag-stimulated pLN we assume that entry into the lymph node and the rate of exit from the lymph node change with the time since cell transfer as $m'_{1i} = m_{1i}e^{\gamma_1 t}$ and $m'_{i1} = m_{i1}e^{\gamma_2 t}$, respectively. The estimated parameters are shown for different rows in the table and in several fits, parameters γ_1 and γ_2 were fixed to 0. Allowing for time-dependent entry/exit rates significantly improved the quality of the model fit to data but resulted in biologically unreasonable predictions (Figure 4). In brackets we show 95% confidence intervals calculated by bootstrapping the residuals with 1000 simulations. Parameters with a star '*' were fixed during the model fit to data.

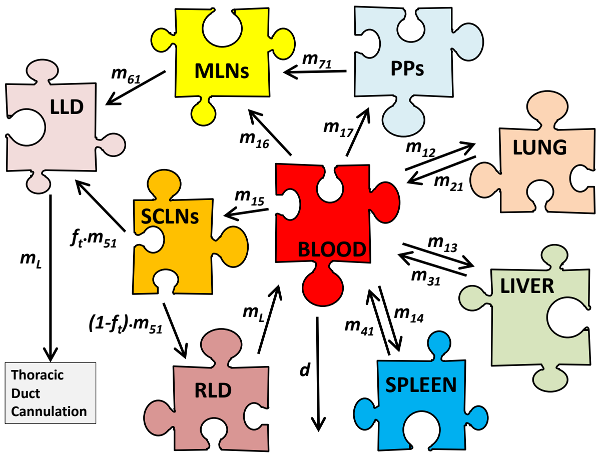

Figure 1: An extension of the basic recirculation model includes explicitly migration of TDLs via the left lymphatic or thoracic duct (LLD) and the right lymphatic duct (RLD). Our calculations suggest that migration of lymphocytes via the thoracic duct is very fast with lymphocytes spending on average 3-4 minutes or less in the duct (see Main text for detail). Assuming that lymphocytes spend substantially longer time in the ducts ($m_L = 2/60 - 1/(2 \times 60)$ min^{-1} or 30 min to 2 hour residence time) did not impact the estimates of the parameters for lymphocyte kinetics. The major change was in the rate of migration to other tissues, d, which was reduced for small values of m_L (results not shown). Including an average 2 hour residence time in the thoracic duct allowed for a better description of the cannulation data with exponentially distributed residence times of TDLs in LNs which was still inferior to that of the model with a gamma-distributed residence times and rapid migration via lymphatic ducts (results not show).

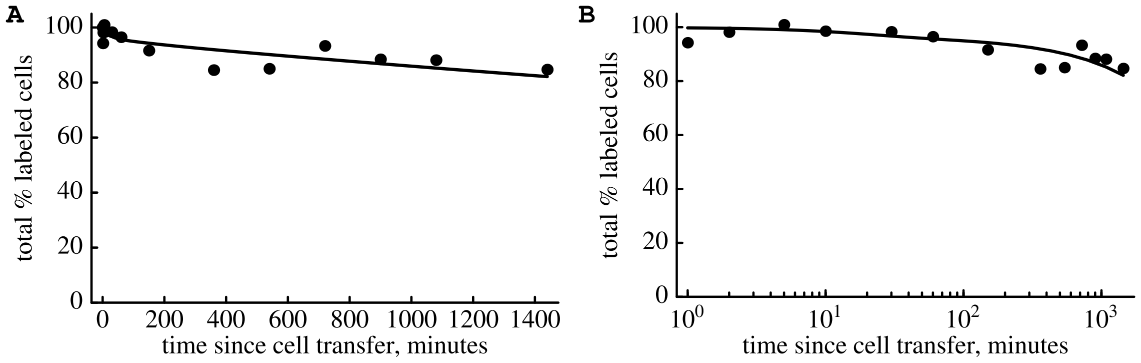

Figure 2: Nearly all transferred TDLs are recovered from 7 major murine tissues over the course of 24 hours. We sum the percent of cells in all organs of rats in the data (points) and plot the prediction of the model (line) on a linear scale (A) or log x-axis (B).

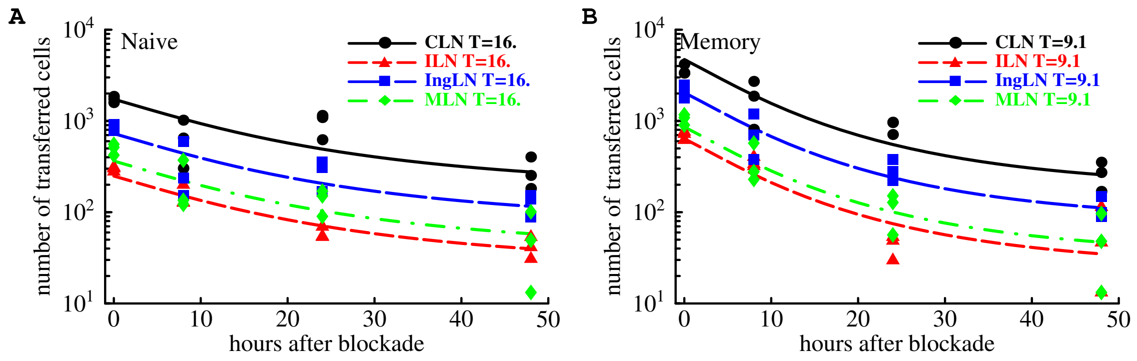

Figure 3: The rate of exit of naive (panel A) and memory (panel B) LCMV-specific CD8 T cells from lymph nodes declines over time following administration of anti-CD62L antibodies. Naive or memory CD8 T cells specific to the GP33 epitope of LCMV were adoptively transferred into naive B6 mice. Twenty four hours later, anti-CD62L antibody was administered that blocks entry of lymphocytes into lymph nodes and Peyer's patches via high endothelial venules (HEVs). The number of transferred CD8 T cells in various lymph nodes after antibody administration was measured over time [1]. Assuming that the antibody prevents lymphocytes from entering lymph nodes from the blood (with 100% efficacy), we fit the mathematical model (eqns. (1)–(6) in Main text) to these experimental data. We let $k = 2$ sub-compartments in every lymph node and assume that transferred lymphocytes reach a steady state prior to antibody treatment. We find that the rate of lymphocyte exit from lymph nodes must decline over time since antibody administration to explain these data ($p = 0.047$ for panel A and $p < 0.001$ for panel B). The estimated rate of decline is $\alpha = 6.7 \times 10^{-4}$ and $\alpha = 8.3 \times 10^{-4}$ min^{-1}, for naive and memory CD8 T cells, respectively. This is in a good agreement with the estimate of $\alpha = 7.6 \times 10^{-4}$ min^{-1} that we found by fitting data on migration of thoracic duct lymphocytes in cannulated rats (Figure 6 in the Main text). The average residence time of naive and memory CD8 T cells estimated by fitting these data is $T = 2/m_{i1} = 16$ hours and 9 hours, for naive and memory CD8 T cells, respectively. If the efficacy of antibodies at blocking entrance of lymphocytes into lymph nodes is not 100%, then the true average residence times are likely to be smaller (Figure 5). Analyzed lymph nodes are: cervical (CLN), iliac (ILN), inguinal (IngLN), and medistinal (MLN) lymph nodes.

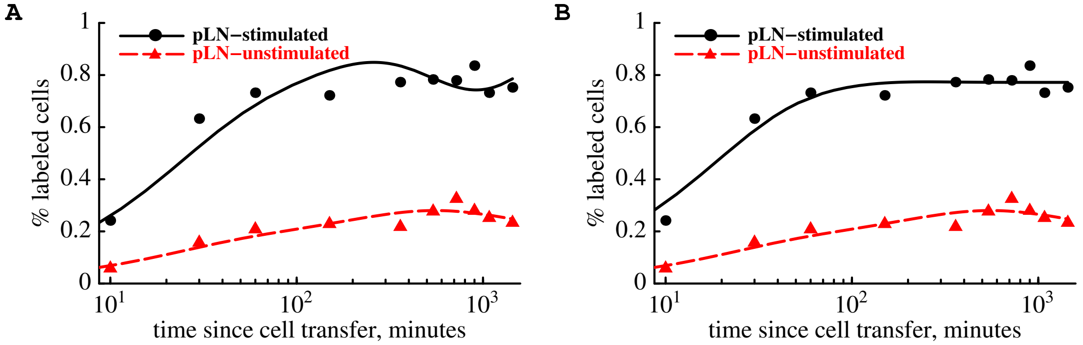

Figure 4: Predictions on TDL accumulation and loss in Ag-stimulated and resting popliteal lymph nodes when rates of entrance into or exit from Ag-stimulated nodes change with time. In panel A we allow entrance rate into Ag-stimulated lymph node to change exponentially with time. Fit of the model predicts that entrance rate of lymphocytes into Ag-stimulated lymph node should increase at a rate $\gamma_1 = 3.2 \times 10^{-4}$ min^{-1} ($m'_{1i} = m_{1i}e^{\gamma_1 t}$; see also Table 1). The fit was statistically better with $\gamma_1 \neq 0$ ($p = 0.004$). However, we believe that this model is biologically unreasonable as it predicts increase in lymphocyte exit rate from Ag-stimulated lymph nodes 1.7 fold (that is, the average residence times of lymphocytes goes from 10.9 hours to 6.6 hours in resting and Ag-stimulated LN (Table 1). This is not biologically reasonable given available data showing that lymphocyte exit rates from antigen-stimulated lymph nodes are not increased [2]. In panel B we allow for both entry and exit rates to change exponentially. The fit predicts that both entry and exit rates should decline with time ($\gamma_1 = \gamma_2 = -9.4 \times 10^{-3}$ min^{-1}, Table 1) resulting in the flat percent of labeled cells in 24 hours post cell transfer with the average residence times of lymphocytes in the Ag-stimulated lymph node being $> 10^6$ hours. Although the fit is significantly better than the fit with constant migration rates ($p = 10^{-6}$), the model prediction is not consistent with experimental data suggesting that 3 days post-infection, the output of lymphocytes from Ag-stimulated lymph nodes is large [2].

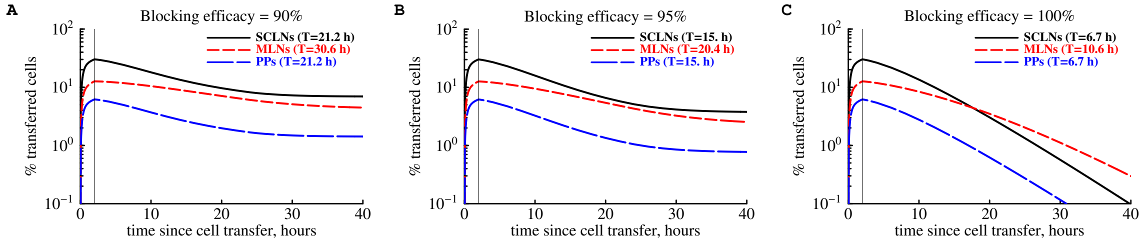

Figure 5: The efficacy of blocking entrance of lymphocytes into lymph nodes via HEVs influences the kinetics of lymphocyte loss from lymph nodes. Following experimental design in [3], we predict the kinetics of the loss of labeled lymphocytes from LNs following blockage of lymphocyte entry into LNs. We use parameters from the Main text (Table 1) and predict the percent of adoptively transferred lymphocytes in various LNs following administration of antibodies blocking entrance of lymphocytes into the LNs 2 hours post-transfer [3]. To simulate the effect of blocking antibodies we let the rate of entrance of lymphocytes into the lymph nodes or Peyer's patches to be reduced to $(1-\epsilon)m_{1i}$ where ϵ is the antibody blocking efficacy ($\epsilon = 90\%$ (panel A), 95% (panel B) and 100% (panel C)) and $i = 5, 6, 7$. We track the percent of transferred lymphocytes in subcutaneous (SCLNs) and mesenteric (MLNs) lymph nodes and Peyer's patches (PPs) over time. Distribution of the residence time in LNs is given by the gamma distribution with the shape parameter $k = 2$. The observed rate at which lymphocyte numbers decline over time, λ, and the estimated average residence time, $T = 1/\lambda$, depend strongly on the blocking efficacy ϵ. We estimate the rate and the average residence time by taking model predictions in panels A-C from 3 to 48 hours in 10 hour intervals and fitting an exponential function to these simulated data. Estimated average residence time T is indicated on individual panels. In the model, the actual average residence time of lymphocytes in lymph nodes is 10 hours (Table 1). A slower decline of lymphocytes in MLNs is due to entrance of lymphocytes from PPs which occurs via afferent lymphatics and we assume that antibody blockage does influence this route of lymphocyte migration.

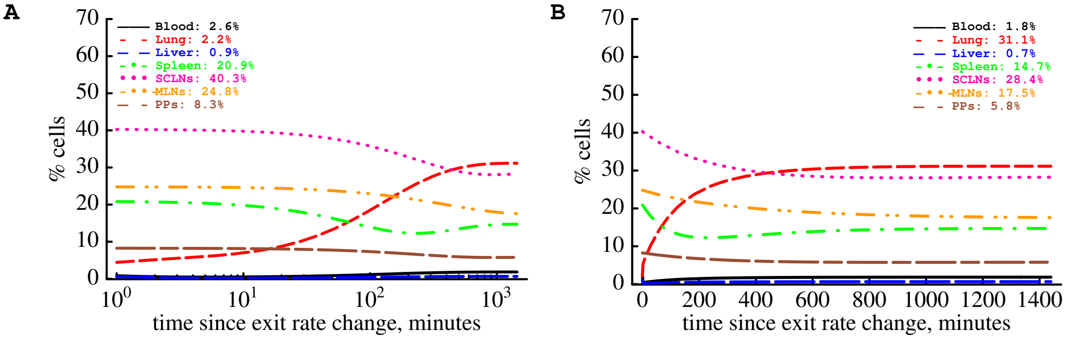

Figure 6: Increase in the average residence time of TDLs in the lung 20 fold leads to a large increase in the number of lymphocytes in the lung. We start at the steady state in the control animal (Table 2 in the Main text and see numbers in panel A). Then we decrease the rate of exit of TDLs from the lung 20 fold (from $m_{21} = 2.24$ min^{-1} to $m_{21} = 0.11$ min^{-1}) and track changes in the percent of TDLs in different organs. The expected steady state of lymphocytes is shown on panel B and in Table 2 in the Main text. The panels show identical dynamics plotted either on the log (A) or linear (B) x-axis.

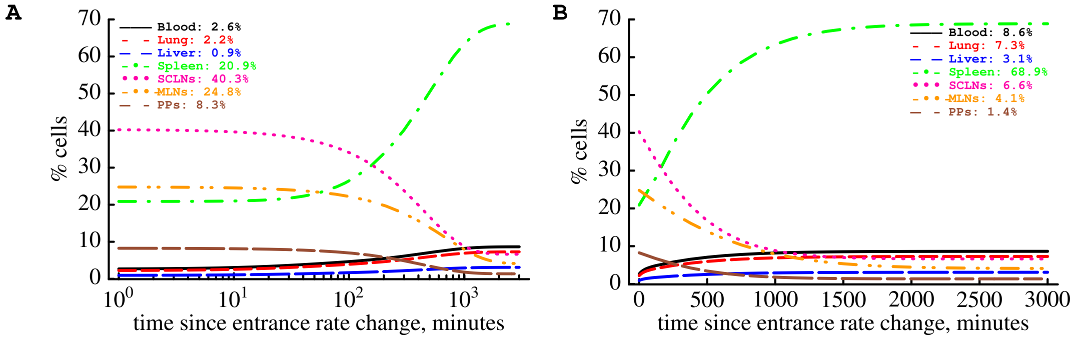

Figure 7: Blocking entrance of TDLs into LNs and Peyer's patches leads to accumulation of lymphocytes in the spleen, lung, and liver. We start at the steady state in the control animal (Table 2 in the Main text and see numbers in panel A). Then we decrease the rate of entrance of TDLs into LNs and PPs 20 fold and track changes in the percent of TDLs in different organs. The expected steady state of lymphocytes is shown on panel B and in Table 2 in the Main text.

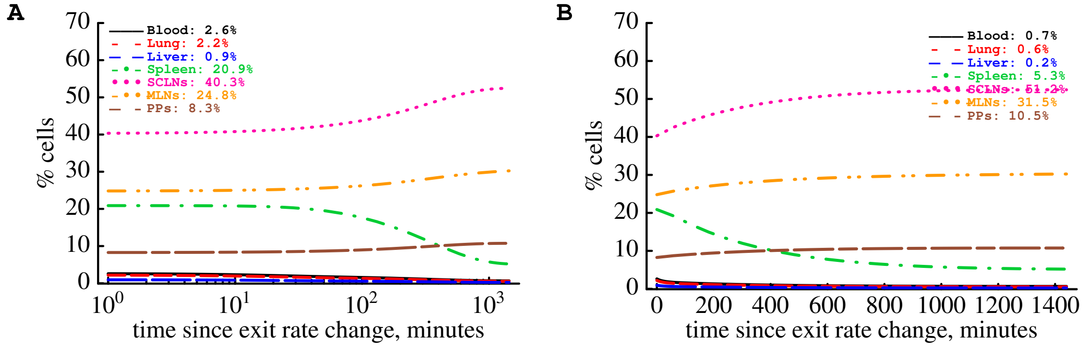

Figure 8: Blocking exit of TDLs from lymph nodes and Peyer's patches leads to a moderate increase of the number of lymphocyte in LNs and lymphopenia in the blood and spleen. We start at the steady state in the control animal (Table 2 in the Main text and see numbers in panel A). We then decrease the rate of exit of TDLs into LNs and PPs 5 fold and track changes in the percent of TDLs in different organs. Value of 5 fold was chosen to match observed changes in lymphocyte numbers in spleens of FTY720-treated hosts [4, 5]. The expected steady state of lymphocytes is shown on panel B and in Table 2 in the main text.

References

1. Harp, J. R., Gilchrist, M. A. & Onami, T. M. 2010 Memory T cells are enriched in lymph nodes of selectin-ligand-deficient mice. *J Immunol*, **185**(10), 5751–5761.

2. Cahill, R. N., Frost, H. & Trnka, Z. 1976 The effects of antigen on the migration of recirculating lymphocytes through single lymph nodes. *J Exp Med*, **143**(4), 870–888.

3. Mandl, J. N., Liou, R., Klauschen, F., Vrisekoop, N., Monteiro, J. P., Yates, A. J., Huang, A. Y. & Germain, R. N. 2012 Quantification of lymph node transit times reveals differences in antigen surveillance strategies of naive CD4+ and CD8+ T cells. *Proc Natl Acad Sci U S A*, **109**(44), 18 036–18 041.

4. Chiba, K., Yanagawa, Y., Masubuchi, Y., Kataoka, H., Kawaguchi, T., Ohtsuki, M. & Hoshino, Y. 1998 FTY720, a novel immunosuppressant, induces sequestration of circulating mature lymphocytes by acceleration of lymphocyte homing in rats. I. FTY720 selectively decreases the number of circulating mature lymphocytes by acceleration of lymphocyte homing. *J Immunol*, **160**(10), 5037–5044.

5. Honig, S. M., Fu, S., Mao, X., Yopp, A., Gunn, M. D., Randolph, G. J. & Bromberg, J. S. 2003 FTY720 stimulates multidrug transporter- and cysteinyl leukotriene-dependent T cell chemotaxis to lymph nodes. *J Clin Invest*, **111**(5), 627–637. doi: 10.1172/JCI16200.
